# Substituent Inductive Effects on the Electrochemical Oxidation of Flavonoids Studied by Square Wave Voltammetry and Ab Initio Calculations

**DOI:** 10.3390/molecules21111422

**Published:** 2016-10-27

**Authors:** Netzahualcóyotl Arroyo-Currás, Víctor M. Rosas-García, Marcelo Videa

**Affiliations:** 1 Departament of Chemistry, Tecnológico de Monterrey, Av. E. Garza Sada 2501 Sur, Monterrey 64849, N.L., Mexico; narroyo@chem.uscb.edu; 2 Facultad de Ciencias Químicas, Universidad Autónoma de Nuevo León, Av. Pedro de Alba S-N Cd. Universitaria, San Nicolás De Los Garza 66451, N.L., Mexico; rosas.victor@gmail.com; 3 School of Engineering and Science, Tecnológico de Monterrey, Av. E. Garza Sada 2501 Sur, Monterrey 64849, N.L., Mexico

**Keywords:** voltammetry, antioxidants, flavonoids, ab initio, electron density distribution

## Abstract

Flavonoids are natural products commonly found in the human diet that show antioxidant, anti-inflammatory and anti-hepatotoxic activities. These nutraceutical properties may relate to the electrochemical activity of flavonoids. To increase the understanding of structure–electrochemical activity relations and the inductive effects that OH substituents have on the redox potential of flavonoids, we carried out square-wave voltammetry experiments and ab initio calculations of eight flavonoids selected following a systematic variation in the number of hydroxyl substituents and their location on the flavan backbone: three flavonols, three anthocyanidins, one anthocyanin and the flavonoid backbone flavone. We compared the effect that the number of –OH groups in the ring B of flavan has on the oxidation potential of the flavonoids considered, finding linear correlations for both flavonols and anthocyanidins ($R^{2}=0.98$). We analyzed the effects that position and number of –OH substituents have on electron density distributions via ab initio quantum chemical calculations. We present direct correlations between structural features and oxidation potentials that provide a deeper insight into the redox chemistry of these molecules.

## 1. Introduction

Flavonoids are a part of the human diet (average daily intake in the U.S. ≈ 1 g/day) [1] and are known to have antioxidant [2,3,4], antihepatotoxic [5,6] and anticancer [7] properties. Flavonoids are polyphenols showing antioxidant capacity; i.e., they act as reducing agents removing free radicals and consequently preventing the oxidation of biologically active molecules. Because of such attributes, they are considered to be nutraceuticals and natural stabilizers [8,9] and have been widely commercialized as dietary supplements. Nevertheless, a comprehensive understanding of the chemistry of flavonoids and their involvement in human metabolism has not been achieved, therefore leaving the true effects of the daily intake of these molecules on the human body to speculation. Moreover, analytical studies regarding the redox chemistry of flavonoids and correlations to structure-activity relationships have not been carried out to the extent of other biomolecules of interest, e.g., quinones [10,11]. We believe that understanding the structural factors that regulate the redox chemistry of flavonoids is essential to the development of quantitative methods that can evaluate their true antioxidant capacity [12], as well as to exploit their potential use as nutraceuticals.

**Figure 1:**
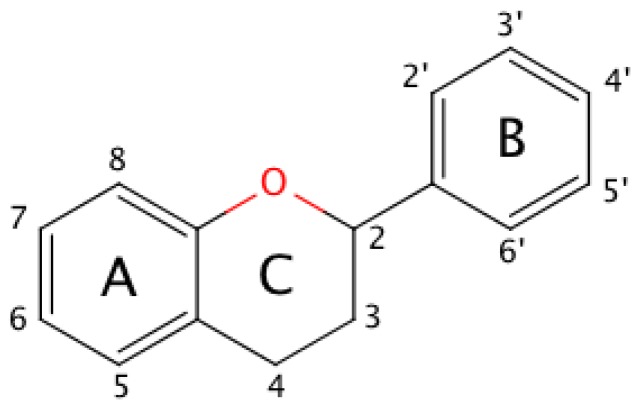
The molecular structure of the flavan nucleus. Substituents present on ring C define families of flavonoids, e.g., a ketone on position 4 yields flavonols, while an oxonium ion in equilibrium with an unsaturated ring gives rise to anthocyanidins.

The basic molecular skeleton of a flavonoid is the flavan nucleus shown in Figure 1 for further reference. Ring C defines different classes of flavonoids: flavanols and anthocyanidins present a pyran ring, whereas flavonols, flavones and flavanones present a pyrone ring [13].

Among the different classes of flavonoids, flavonols and anthocyanidins are the two most frequently found in the human diet, at concentrations up to ∼400 μg/g produce [14,15]. The ubiquitous presence of these molecules in nature as well as the medicinal properties attributed to them have made of flavonols and anthocyanidins the subject of research for several decades. Of particular importance is the determination of their antioxidant capacity by analytical means because of their implication in human health (for example, fighting back oxidative stress). Direct methods study the electronic features of flavonoids as a function of pH or analyte concentration, like the 2,2-diphenyl-1-picrylhydrazyl (DPPH) protocol [16]. Indirect methods measure the radical sequestration power of flavonoids, like the oxygen-radical absorbance capacity test (ORAC) [17]. However, both types of methods provide inconclusive results when comparing the antioxidant capacity of two or more flavonoids under the same conditions [18]. In a study by Csepregi et al. [19], a comparison of the total antioxidant capacity (TAC) evaluated using three different in vitro assays, Trolox Equivalent Antioxidant Capacity (TEAC), Ferric Reducing Antioxidant Potential (FRAP) and DPPH of over 37 phenolic compounds, presented strong evidence that the antioxidant activity of these compounds is dependent on the choice of assay and concludes, based on a thorough statistical analysis, that the results provided by each assay depend on specific features of the chemical structure of such compounds. Excellent reviews about the different methods available to evaluate antioxidant capacity can be found in the literature [20]. Among these, electrochemical techniques have been recognized as the natural tools to evaluate the antioxidant activity of phenolic compounds [21,22,23,24] and have been successfully applied to evaluate the total activity of antioxidants present in foodstuffs such as edible oils, herbs, wine and fruit infusions [25,26,27,28]. In this context, instructive correlations between antioxidant activity and electrochemical parameters have been reported [29,30].

In the present work, we used square wave voltammetry (SWV) [31,32,33,34] to study the first oxidation potential of eight different flavonoids dissolved in anhydrous acetonitrile. We discuss our results with the aid of ab initio quantum mechanical calculations of the molecular orbital energies and electron density distributions on the flavan backbone that contribute to the analysis of the electronic effect of substituents on the antioxidant ability. Importantly, to find relations between oxidation potentials and the substitutional modification of flavonoids, we selected flavonols with a systematic variation in the number of hydroxyl substituents and their location on the flavan backbone (Table 1). In order to extend structural comparisons, we also selected anthocyanidins with a similar approach and one anthocyanin with a glucosidic bond in position 3 of ring C. Since the electrochemical oxidation of flavonoids is pH dependent [35], we selected an aprotic solvent to avoid any pH-related effects in our electrochemical determinations.

## 2. Results

### 2.1. General Relationships between Structure and Oxidation Potential

**Figure 2:**
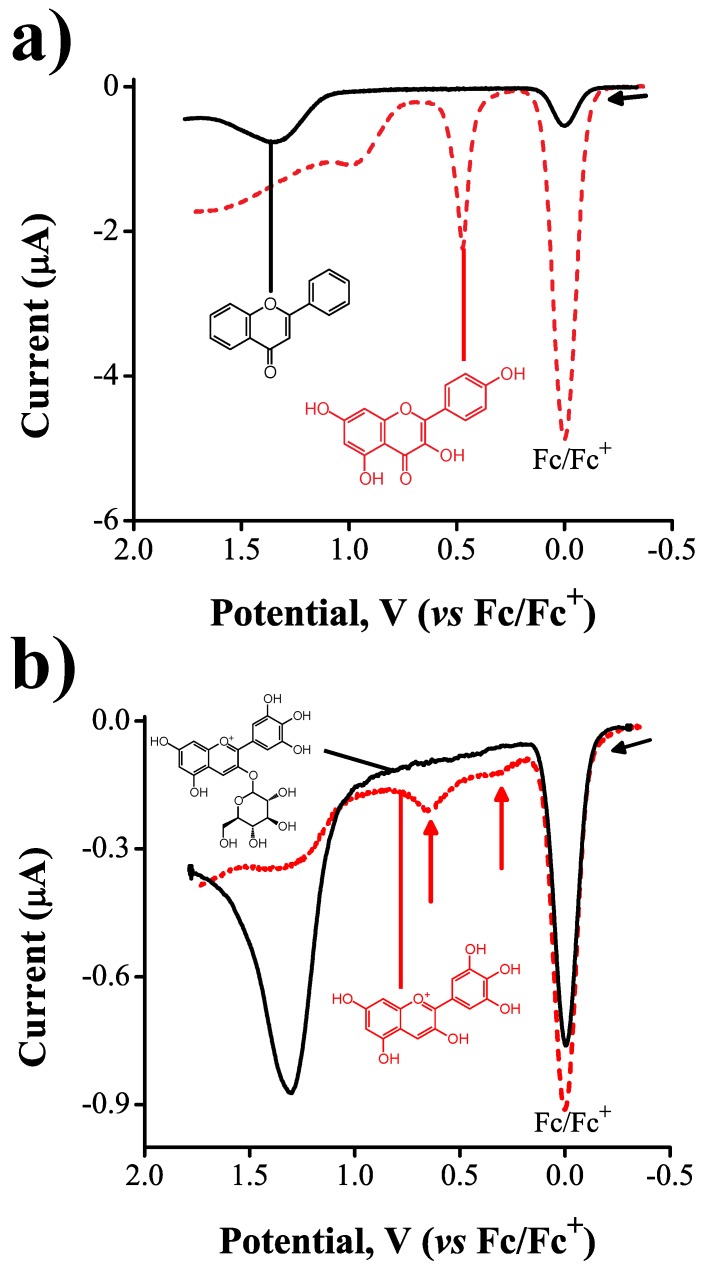
(**a**) square wave voltammograms (Ψ vs. *E*) of 0.5 mM flavone + 0.1 mM ferrocene + 0.1 M tetraethyammonium tetrafluoroborate, TEATFB (solid line) and 2.5 mM kaempferol + 0.5 mM ferrocene + 0.1 M TEATFB (dashed line); and (**b**) square wave voltammograms of 0.5 mM delphinidin-3*O*-glucoside + 0.09 mM ferrocene + 0.1 M TEATFB (solid line) and 0.1 mM delphinidin + 0.11 mM ferrocene + 0.1 M TEATFB (dashed line). In all experiments ΔE=50 mV, f=1 Hz.

We carried out experiments to understand the extent to which the electrochemical activity of flavonoids is influenced by the electron–donor effect of OH substituents present in the flavan backbone or from the oxidation of ring C. A good insight into the electrochemical response was obtained by comparing the square wave voltammograms of flavone and kaempferol. These molecules share the same flavone backbone, but kaempferol has four extra hydroxyl substituents (see Table 1, Figure 2a).

In the voltammograms, the first reversible wave corresponds to the oxidation of Fc (used as the internal standard). Flavone presented one irreversible oxidation wave at $E_{0}^{\prime }$ = 1.3 V, but no other processes were observed between 0.00 V and 1.30 V (solid line). Kaempferol, on the other hand, presented a clear first oxidation wave at a lower potential $E_{0}^{\prime }$ = 0.46 V, followed by a second oxidation process at $E_{0}^{\prime }\approx$ 0.95 V (dashed line).

The first oxidation wave of kaempferol in acetonitrile was previously studied by Jørgensen and coworkers [35] using bulk electrolysis coupled to liquid chromatography-mass spectrometry (LC-MS). They attributed such a redox process to the successive oxidations of the hydroxyl groups present in positions 4${}^{\prime }$ and 3. They also determined that the one-electron oxidation of the 3OH substituent in kaempferol is the rate-determining step. Theoretical calculations of electron density distribution on flavonoids have come to the same conclusion [36]. In the case of flavone, the fact that no redox processes occurred before 1.30 V vs. Fc|Fc${}^{+}$ can be explained by the absence of hydroxyl substituents present in its chemical structure. Furthermore, the redox process observed at 1.30 V is attributed to the irreversible oxidation of ring C with subsequent rearrangement of the flavan backbone [35].

To determine whether other classes of flavonoids, like anthocyanidins, shared similar reactivity in the 3OH substituent, we carried out experiments to compare the electrochemical behavior of delphinidin and delphinidin-3*O*-glucoside (Figure 2b). Both delphinidin and its 3*O*-glucoside share the same backbone, but in the latter, the substituent 3OH is blocked by an ether bond to the carbohydrate. While the voltammograms of delphinidin presented two oxidation waves at $E_{0}^{\prime }$ = 0.29 V and $E_{0}^{\prime }$ = 0.64 V, indicated by arrows in Figure 2b, the voltammograms corresponding to delphinidin-3*O*-glucoside were free of faradaic processes up to potentials of 1.25 V, behavior similar to that of flavone. The presence of the ether bond blocked the reactivity of delphinidin at the 3OH location, presumably due to redistribution of the electron density of the neutral molecule. These results are an experimental confirmation of the relevance of the 3OH substituent as a key reactive site during the electrochemical oxidation of flavonols. The anodic peak observed in the voltammograms of delphinidin-3*O*-glucoside at $E_{0}^{\prime }$ = 1.25 V may also be attributed to the oxidation of ring C of the flavan backbone as in the case of flavone (Figure 2a). The fact that no electrochemical processes are observed before 1.25 V demonstrates that, although delphinidin-3*O*-glucoside has a total of five OH substituents in the flavan backbone, its electrochemical activity is blocked by the impossibility of ionization of substituent 3OH.

### 2.2. Inductive Effects on Oxidation Potentials

**Figure 3:**
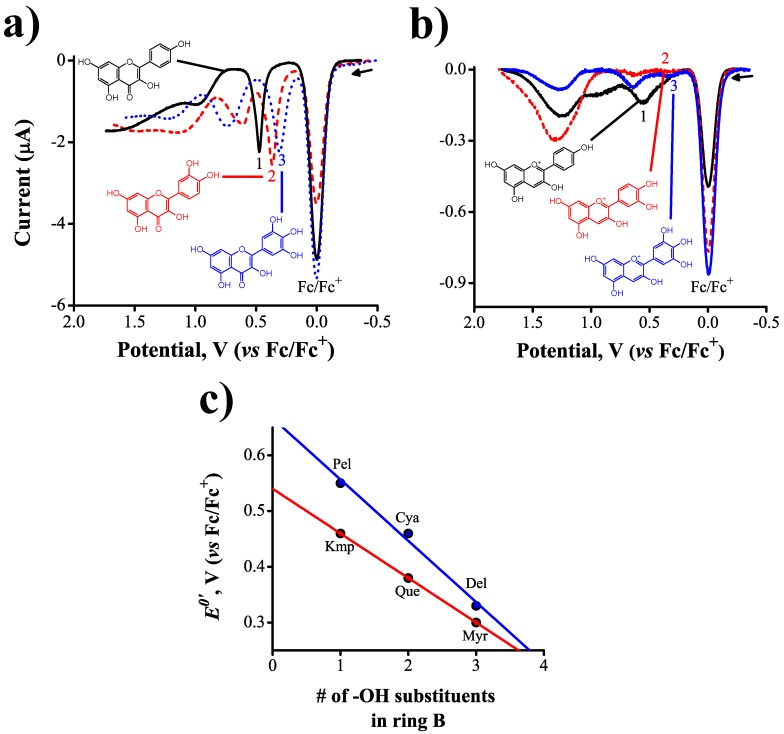
(**a**) square wave voltammograms (Ψ vs. *E*) of 0.05 mM kaempferol (1), quercetin (2), and myricetin (3) in 0.5 mM ferrocene + 0.1 M TEATFB; (**b**) square wave voltammograms of 0.01 mM pelargonidin (1) in 0.25 mM ferrocene + 0.1 M TEATFB, cyanidin (2) in 0.4 mM ferrocene + 0.1 M TEATFB, and delphinidin (3) in 0.5 mM ferrocene + 0.1 M TEATFB, ΔE=50 mV, f=1 Hz. The numbers indicate how many substituents are present in ring B of the corresponding flavonoid; and (**c**) linear correlations between formal oxidation potential and number of OH substituents in the ring B of flavonoids.

To demonstrate that the oxidation potentials of flavonoids are susceptible to changes in the electron density distribution across the conjugated flavan backbone, we carried out voltammetry experiments with three molecules from each one of two different classes of flavonoids: flavonols and anthocyanidins. The flavonoids selected differed from each other in the number of hydroxyl substituents present in their ring B (see Table 1 for clarity). Figure 3a,b show the square wave voltammograms obtained. Each substituent bonded to the flavan structure has an inductive effect on the net distribution of charge densities, with identical substituents having additive effects.

The voltammograms presented in Figure 3 show irreversible electron transfer oxidations for all flavonoids studied. We observed a clear linear correlation between the oxidation potential of the flavonoids and the number of hydroxyl substituents present in their ring B (Figure 3c). A summary of formal reduction potentials, $E_{0}^{\prime }$, extracted from such voltammograms is shown in Table 2.

Anthocyanidins were harder to oxidize than flavonols due to the presence of the oxonium ion in ring C, in contrast with the more electron-rich oxygen–ether present in flavonols. The correlation coefficients obtained in the regression analysis were *R*${}^{2}$ = 0.98 for both flavonols and anthocyanidins. Furthermore, the slope obtained with anthocyanidins show a larger dependence between the formal reduction potentials, $E_{0}^{\prime }$ and the number of hydroxyl substituents in ring C, going from pelargonidin (0.55 V vs. Fc/Fc${}^{+}$) to delphinidin (0.33 V vs. Fc/Fc${}^{+}$). A comparison of the substituent effects in anthocyanidins and flavonols can be drawn from the ratio of the slopes in Figure 3c, which is $m_{\mathrm{anthocyanidin}}/m_{\mathrm{flavonol}}=1.4$, a ratio that arises from the structural differences in ring C, accounting for a difference of 100 mV between the reduction potentials of pelargonidin and kaempferol. Additionally, these results indicate that flavonols are slightly better antioxidant agents than anthocyanidins because they have lower oxidation potentials at larger number of OH substituents in ring C. We have shown, for the first time across multiple classes of flavonoids, that the oxidation potentials in these families of molecules are directly related to their substitutional chemistry, an important observation that could be exploited in the development of a new antioxidant activity scale.

### 2.3. Orbital Energy and Electron Density Considerations

In order to have a better insight into the aforementioned structural trends, quantum chemical calculations were performed to investigate the effect that the presence of different substituents has on the electron density distribution of each flavonoid considered. In the cases of kaempferol, quercetin and myricetin, our global minima turned out to be equal to those reported by Aparicio [37]. We used the mechanism proposed by Webster [38] for the electro-oxidation of vitamin E to guide the construction of intermediates for the flavonoids of interest.

**Figure 4:**
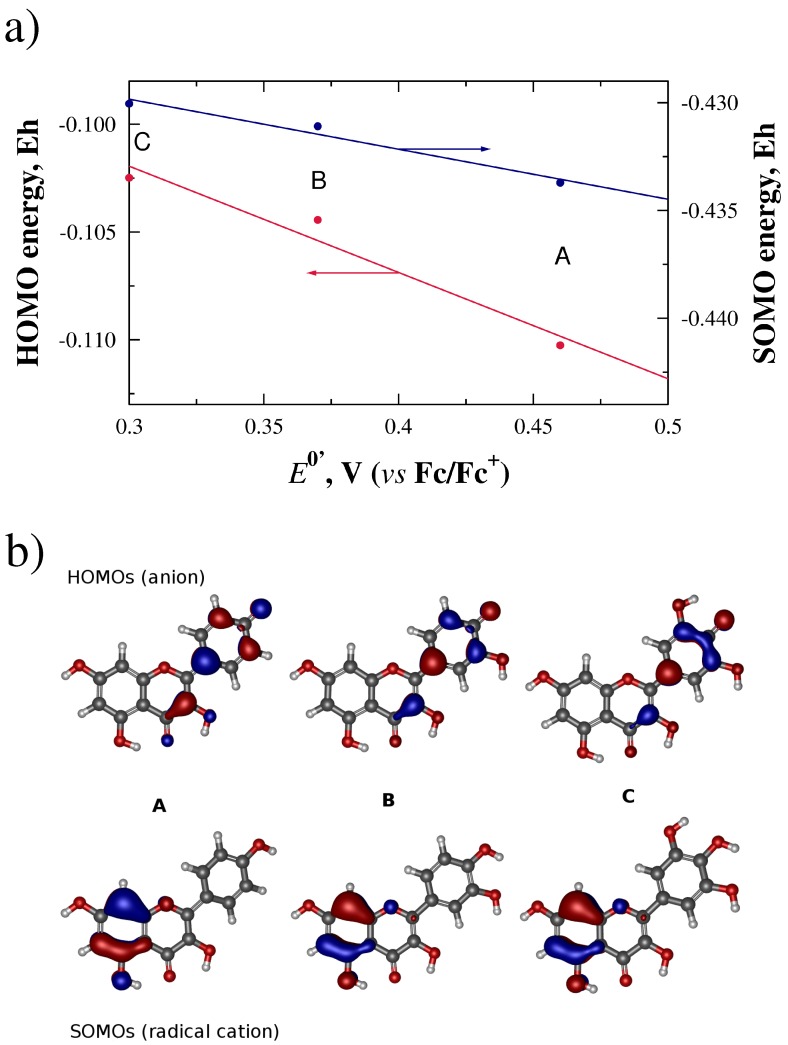
(**a**) linear correlations between $E_{0}^{\prime }$ potentials, in Volts, and molecular orbital energies for the HOMO of the deprotonated flavonols (anion) and the SOMO for the radical cation, in Hartrees; (**b**) HOMOs and SOMOs for flavonols kaempferol (A), quercetin (B), and myricetin (C) obtained at the HF/SVP level of theory.

After the geometry optimizations, we attempted correlations between the potentials and the orbital energies. For the flavonols, linear regressions with values of *R*${}^{2}$ close to 1 were obtained when correlating the reduction potentials to the HOMO of the anion (*R*${}^{2}$ = 0.958), and to the SOMO of the cationic radical (*R*${}^{2}$ = 0.971) as shown in Figure 4a. We then visualized the orbitals which yielded the best energy-potential correlations. From Figure 4b, we can observe that the HOMO of the anionic species for kaempferol (A), quercetin (B) and myricetin (C), span mainly rings B and C. This orbital involves both the 4${}^{\prime }$ OH and the 3 OH substituents, as previously suggested by the results of Heijnen et al. [36]. On the other hand, the SOMO seems to be restricted to ring A.

**Figure 5:**
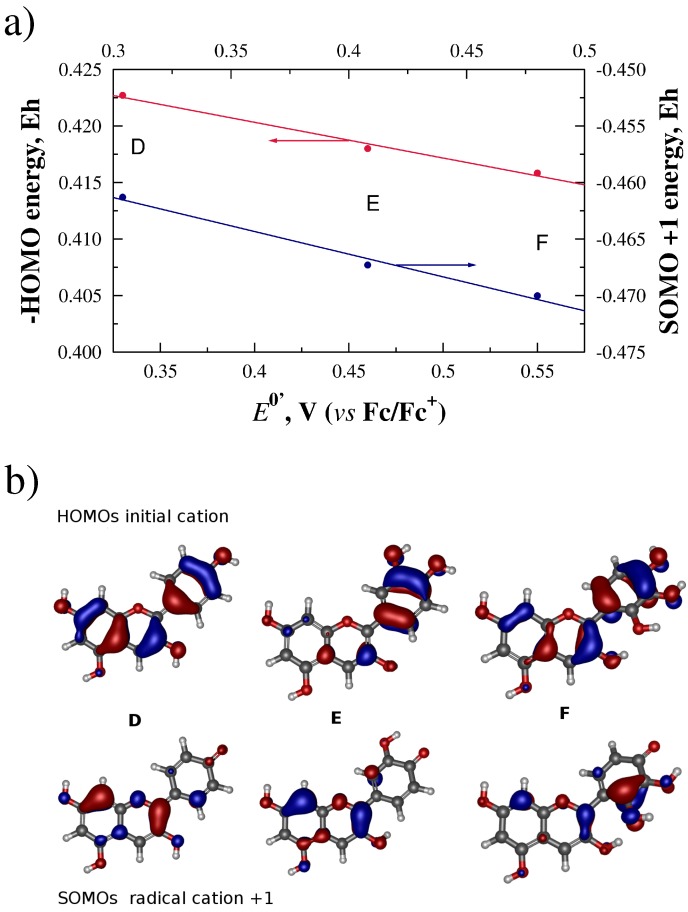
(**a**) linear correlations between $E_{0}^{\prime }$ potentials, in volts, HOMO and SOMO orbital energies of radical anions, in Hartrees. The sign of the HOMO energies were reversed to plot trends with similar slopes; and (**b**) HOMOs for cationic anthocyanidins and SOMOs for radical cation +1 for pelargonidin (D), cyanidin (E), and delphinidin (F), calculated at the HF/SVP level of theory.

In contrast, anthocyanidins showed reduction potentials with a good correlation coefficient *R*${}^{2}$ to the HOMO of the initial cationic form (*R*${}^{2}$ = 0.989), and to the SOMOs of the +1 radical cation (*R*${}^{2}$ = 0.988) (see Figure 5a). Visualization of the orbitals involved, presented in Figure 5b, shows that the HOMOs of the initial species span all three rings A, B and C, even in the case of delfinidin (F), where the C ring is twisted out of the plane of the A–B ring system. Also in Figure 5b, the SOMO of the radical species seems to span rings A and B in the case of pelargonidin (D) and cyanidin (E), while in delfinidin (F), there is increased contribution of π orbitals from ring C in this orbital. This increased contribution coincides with the presence of the OH substituents on ring C closer to ring B.

## 3. Discussion

We demonstrate here the ability of square wave voltammetry coupled to ab initio calculations to reveal structure–electrochemical activity relations potentially related to the antioxidant activity of flavonoids. To this end, we determined the first oxidation potential of several flavonoids from two different structural classes, flavonols and anthocyanidins, as a function of three structural modifications: (1) the presence or absence of -OH substituents in the flavan backbone; (2) blockage of the 3OH substituent in ring C to determine its importance as the rate-limiting step for the oxidation reaction; and (3) the effect that serially increasing the number of -OH substituents in ring B has on the first oxidation potential of these molecules. Our measurements reveal that the redox activity of flavonols and anthocyanidins arises mainly from the hydroxyl substituents present on the flavan backbone, with some activity arising from ring C at very positive potentials, for example 1.2 V in the case of flavone. In fact, the absence of -OH substituents causes a disappearance of the oxidation wave seen at 0.46 V for kaempferol, a molecule that has the same backbone as flavone (Figure 2a). Of the various -OH substituents found in flavonols and anthocyanidins, the one found in position 3 of the flavan backbone was reported by Jørgensen et al. [35] and Heijnen et al. [36] to act as the rate limiting step in the oxidation of flavonoids. We confirmed the importance of this group using the anthocyanidin delphinidin as an example: by blocking the 3OH substituent via formation of an ether bond to a carbohydrate, i.e., delphinidin-3*O*-glucoside, the oxidation process at 0.33 V disappears (Figure 2b). If the 3OH substituent is maintained in the structure, the reactivity of flavonols and anthocyanidins becomes then a function of the substitutional chemistry of ring B. The oxidation potentials of both classes of flavonoids decrease monotonically with increasing number of –OH substituents in ring B, by about 80–90 mV. The anthocyanidins are, overall, harder to oxidize than their analogous flavonols as indicated by more positive oxidation potentials, an effect presumably arising from differences in structural features and electron density distributions in ring C of these molecules—for example, the presence of the oxinium ion in anthocyanidins. This effect, however, becomes less important as the number of OH substituents in ring B increases (myricetin and delphinidin oxidize at similar potentials).

**Figure 6:**
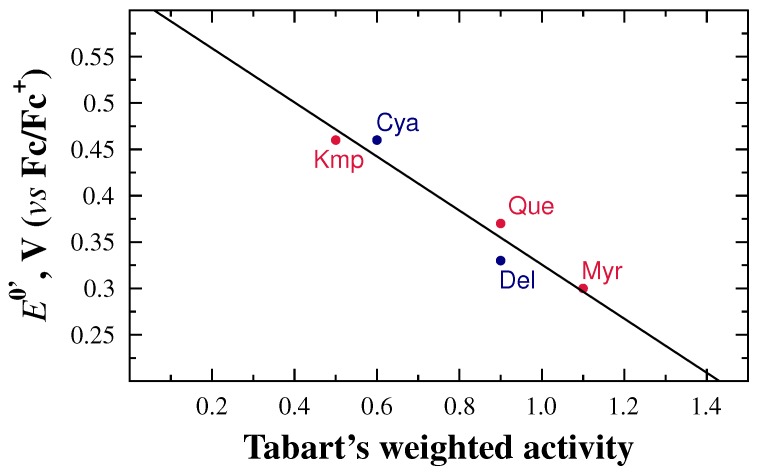
Correlation between formal reduction potentials, $E_{0}^{\prime }$ and Tabart’s antioxidant weighted average activity.

The overreaching goal of our work was to demonstrate the value of electrochemical parameters, such as the first oxidation potential, as a metric of antioxidant activity in flavonoids. To demonstrate this, we constructed a plot of $E_{0}^{\prime }$ vs. values of antioxidant activity extracted from the work of Tabart et al. [18]. In their study, Tabart et al. used four different approaches to measure antioxidant activity: DPPH or TEAC (reducing capacity), ORAC (peroxyl radical scavenging capacity), haemolysis (protection of a biological sample), and ESR (free radical evaluation). They standardized the values of each assay by measuring the antioxidant activity of the antioxidant Trolox, used as reference. Finally, they proposed calculating a global antioxidant capacity as a weighted average of the results obtained by the DPPH, ORAC, resistance to haemolysis, and ESR assays. Figure 6 shows the result of plotting the oxidation potentials obtained in this work vs. the standardized, weighted-averaged values reported by Tabart et al. Although the $E_{0}^{\prime }$ values are not standardized in any way, a clear linear trend is observed with $R^{2}=0.99$. These results strongly emphasize the significance the electrochemical parameter $E_{0}^{\prime }$ in the determination of antioxidant activity of flavonoids towards the construction of a global antioxidant activity scale.

**Figure 7:**
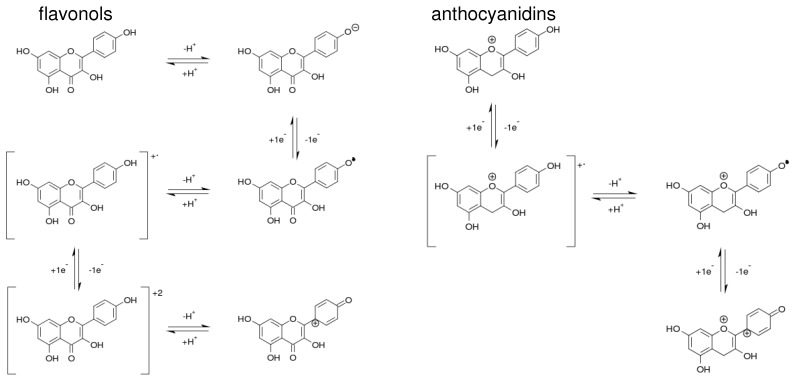
Proposed oxidation mechanisms for flavonols and anthocyanidins.

From a theoretical perspective, our ab initio calculations suggest that the oxidation of flavonoids and of anthocyanidins proceed through two different mechanisms. By putting together the different correlations of the orbital energies to the measured potentials, we developed a picture of two different mechanisms (see Figure 7), which are consistent with the fact that the redox activity of these two flavonol groups is different as shown in Figure 3c and the correlations between redox potentials and the energies of the molecular orbitals shown in Figure 4a and Figure 5a.

Flavonoids start by losing a proton in a OH substituent in ring B, yielding an anion. This anion is then oxidized to a neutral radical and then deprotonated to a radical cation. The radical cation is further oxidized to a +2 charged cation that is then deprotonated to the singly charged, oxidized species. In contrast, anthocyanidines start by losing an electron, forming a +2 charged radical cation. This cation loses a proton to yield a singly charged cationic radical, which is further oxidized by losing one electron to produce the final oxidized species. The two mechanisms differ in the existence of a fast protonation–deprotonation equilibrium involved in the oxidation of flavonoids to facilitate the loss of one electron to generate a radical cation, while anthocyanidins directly oxidize to a radical cation. In both cases, once the radical cation forms, another electron and a proton are lost to generate the final oxidized species.

## 4. Materials and Methods

### 4.1. Chemicals and Solutions

Quercetin, kaempferol, myricetin, pelargonidin chloride, delphinidin chloride, cyanidin chloride, delphinidine-3*O*-glucoside and flavone (chemical structures shown in Table 1) were purchased from Sigma Aldrich (HPLC grade, St. Louis, MO, USA) and used without additional purification. Anhydrous acetonitrile (CH${}_{3}$CN, Sigma Aldrich, 99.8%) was distilled from phosphorous pentoxide prior to each experiment. Tetraethylammonium tetrafluoroborate (TEATFB, Sigma Aldrich) was used as received. Stock solutions of 0.1 M TEATFB in CH${}_{3}$CN were prepared and stored in a desiccator. Ferrocene (Fc) was used as internal standard at concentrations between 0.025–0.1 mM in stock solution. Solutions of 0.01–0.5 mM flavonoid in stock + Fc were prepared fresh prior to every measurement.

### 4.2. Instrumentation

Square wave voltammetry experiments were carried out using a potentiostat/galvanostat PAR-M273A (EG & G Princeton Applied Research, Oak Ridge, TN, USA). The ohmic drop, $iR_{u}$, was corrected using positive feedback with input of 210 Ω. A three-electrode cell was used with a glassy carbon disk of 1 mm of diameter (geometric area $7.8\times 10^{-3}$ cm${}^{2}$) as the working electrode. The surface of the working electrode was polished with 0.1 μm diameter basic alumina suspension (AP-D Suspension, Struers ApS, Ballerup Denmark) and washed with acetone prior to every experiment. A platinum mesh was used as the counter electrode (ca. 1 cm${}^{2}$). Potentials were measured against a solid platinum pseudo-reference electrode and are reported vs. the ferrocinium/ferrocene (Fc${}^{+}$/Fc) couple, following recommendations by the International Union of Pure and Applied Chemistry (IUPAC) [39].

### 4.3. Ab Initio Calculations

A full conformational search was done on all the flavonoids and antocyanidins. Thirty-two conformers were optimized for kaempferol, 64 for quercetin and 128 for myricetin, while for the anthocyanidins, 16 were optimized for pelargonidin, 64 for cianidin, and 128 for delfinidin. Duplicate structures were eliminated, and all the unique extrema obtained were characterized as minima by a vibrational analysis that showed only real frequencies. The global minimum for each compound was selected to generate the diverse intermediates of a mechanism analogous to the one proposed by Webster [38] for the electro-oxidation of vitamin E. All the intermediates were geometry-optimized and their vibrational frequencies checked. This resulted in four intermediates for each flavonol/anthocyanidin in addition to the final oxidized species. Molecular structures were constructed using Avogadro [40] ab initio calculations (both optimizations and single points) employed ORCA v. 2.8 (Frank Neese and Frank Wennmohs, Bonn, Germany) [41]. All single points, geometry optimizations and vibrational analyses were carried out at the Hartree-Fock level of theory with a Karlsruhe Split Valence Polarization basis set (HF/SVP) [42]. Orbitals were visualized by means of Gabedit v. 2.0 (Abdul-Rahman Allouche, Villeurbanne, France) [43] and rendered in POVRay v. 3.7 (Persistence of Vision Pty. Ltd., Williamstown, Victoria, Australia) [44].

## 5. Conclusions

The overreaching goal of our work was to demonstrate the value of electrochemical parameters, such as the first oxidation potential, as a metric of antioxidant activity in flavonoids. To achieve this, we measured the first oxidation potential of three flavonols and three anthocyanidins in acetonitrile, and studied the changes in such oxidation potentials caused by substituent inductive effects. We explain the changes in reactivity observed with the aid of ab initio calculations and confirm previous results related to the reactivity of these molecules. Finally, we demonstrate how the oxidation potentials determined in this work linearly correlate with values of antioxidant activity previously reported, demonstrating with this the value of electrochemical parameters as a quantitative metric of antioxidant power for molecules like flavonoids.

## Figures and Tables

**Table 1:** List of flavonoids by name, chemical structure. The structures shown are ordered according to the number of hydroxyl substituents present in ring B.

Compounds	Structure
**Flavonols**	
kaempferol	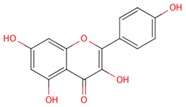
quercetin	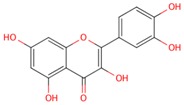
myricetin	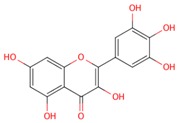
**Anthocyanidins**	
pelargonidin	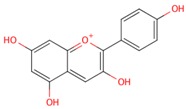
cyanidin	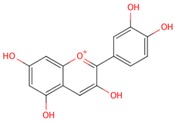
delphinidin	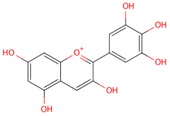

**Table 2:** Table of extent of substitution in B ring, oxidation potentials $E_{0}^{\prime }$ and HOMO and SOMO orbital energies in electron-volts (eV) for flavonols and anthocyanidins.

**Flavonols**	**OH**	$E_{0}^{\prime }$/V	**HOMO**	**SOMO**	
			**Anion**	**Cationic Radical**	
kaempferol	1	0.46	−3.0002	−11.8019	
quercetin	2	0.37	−2.8419	−11.7306	
myricetin	3	0.30	−2.7887	−11.7022	
**Anthocyanidins**	**OH**	**$E_{0}^{\prime }$/V**	**HOMO**	**SOMO+1**	**SOMO+2**
			**Cation**	**Cationic Radical**	**Cationic Radical**
pelargonidin	1	0.55	−11.3155	−12.7898	−15.8675
cyanidin	2	0.46	−11.3743	−12.7160	−15.8230
delphinidin	3	0.33	−11.5025	−12.5528	−15.6110

## Abbreviations

The following abbreviations are used in this manuscript:

DPPH: 2,2-diphenyl-1-picrylhydrazyl
ESR: Electron Paramagnetic Resonance
Fc: ferrocene
FRAP: ferric reducing antioxidant potential
HOMO: highest occupied molecular orbital
HF: Hartree–Fock
ORAC: oxygen-radical absorbance capacity
SOMO: single occupied molecular orbital
SVP: Karlsruhe split valence polarization basis set
SWV: square wave voltammetry
TAC: Total antioxidant capacity
TEAC: Trolox equivalent antioxidant capacity
TEATFB: tetraethylammonium tetrafluoroborate
Cya: cyanidin
Del: delphinidin
Kmp: kaempferol
Myr: myricetin
Pel: pelargonidin
Que: quercetin
